# Cervical cancer and the global health agenda: Insights from multiple policy-analysis frameworks

**DOI:** 10.1080/17441692.2013.850524

**Published:** 2013-11-18

**Authors:** Justin O. Parkhurst, Madhulika Vulimiri

**Affiliations:** a Department of Global Health and Development, London School of Hygiene and Tropical Medicine, London, UK; b Department of Health Policy and Management, University of North Carolina, Chapel Hill, NC, USA

**Keywords:** cervical cancer, global health policy, policy analysis, non-communicable diseases, priority setting

## Abstract

Cervical cancer is the second leading cause of cancer deaths for women globally, with an estimated 88% of deaths occurring in the developing world. Available technologies have dramatically reduced mortality in high-income settings, yet cervical cancer receives considerably little attention on the global health policy landscape. The authors applied four policy-analysis frameworks to literature on global cervical cancer to explore the question of why cervical cancer may not be receiving the international attention it may otherwise warrant. Each framework explores the process of agenda setting and discerns factors that either facilitate or hinder policy change in cases where there is both a clear problem and a potential effective solution. In combination, these frameworks highlight a number of crucial elements that may be needed to raise the profile of cervical cancer on global health agendas, including improving local (national or sub-national) information on the condition; increasing mobilisation of affected civil society groups; framing cervical cancer debates in ways that build upon its classification as a non-communicable disease (NCD) and an issue of women's rights; linking cervical cancer screening to well-funded services such as those for HIV treatment in some countries; and identifying key global policy windows of opportunity to promote the cervical cancer agenda, including emerging NCD global health discussions and post-2015 reviews of the Millennium Development Goals.

## Introduction

According to recent estimates, approximately 500,000 women were diagnosed with cervical cancer in 2008, with 274,000 estimated attributable deaths (Ferlay et al., 2010). This figure is similar to the 287,000 deaths from maternal causes estimated in 2010 by the World Health Organisation (WHO; World Health Organisation, 2012). As with maternal mortality, a vast majority of these deaths affect women in low-income countries, with 88% of cervical cancer deaths estimated to occur in these areas (Ferlay et al., 2010). In December 2012, the group Cervical Cancer Action published a ‘Report Card’ that drew on WHO projections to state: ‘[B]y 2030, cervical cancer is expected to kill over 474,000 women per year and over 95% of these deaths are expected to be in low- and middle-income countries. In sub-Saharan Africa alone, cervical cancer rates are expected to double’ (p. 6).

Gostin (2012) has argued that ‘[h]ealth inequalities – the inequitable distribution of disease and early death between the rich and poor – represent perhaps the most enduring and consequential global health challenge’ (Gostin, 2012, p. 2087). Yet, while the reduction of maternal mortality has been enshrined as a global policy priority in the Millennium Development Goals, presumably due to the incredible inequity of disease burden, the reduction of cervical cancer mortality has not yet been able to achieve this same status. Similarly, Louie, de Sanjose, and Mayaud (2009) reviewed the epidemiological literature on the burden of cervical cancer in Africa and concluded that ‘[t]he magnitude of the problem has been under-recognised and under-prioritised compared to competing health priorities such as HIV/AIDS, tuberculosis and malaria’ (p. 1287).

This stated ‘under-prioritisation’ is not due to an inability to prevent or treat cervical cancer. The overwhelming proportion of deaths occurring in low-income countries is a testament to the efficacy of key interventions that have prevented cervical cancer or reduced mortality in high-income settings. The Pap smear has been credited with significantly lowering cervical cancer mortality rates in developed countries in the 1960s and 1970s, and since then, cytologic screening methods have further improved to effectively detect and treat precancerous lesions (Sankaranarayanan, Budukh, & Rajkumar, 2001). Questions may be raised about the affordability of both screening and treatment in low-income settings, which will have limited health resources and infrastructure. Yet, in the last decade, visual inspection with acetic acid (VIA) or visual inspection with Lugol's iodine (VILI) has been validated as a cost-effective and accurate screening tool for resource-constrained settings (Sauvaget, Fayette, Muwonge, Wesley, & Sankaranarayanan, 2011). Moreover, cryotherapy, which removes precancerous tissue from the cervix, has also been found to be a cost-effective treatment for low- and middle-income countries (Reeler et al., 2009), and it has been embraced by the WHO (2009a). Treatment of established or advanced cancer may indeed prove difficult in resource-limited settings, but VIA and cryotherapy provide much more feasible options that could avert a large number of cancer cases in the first place (the WHO notes the difficulty in predicting exact numbers of cases averted but concludes that the potential benefit is very high despite difficulty predicting adverse outcomes, recommending the treatment in low-, middle-, and high-income settings; WHO, 2011b).

Furthermore, recent introduction of vaccines for the human papillomavirus (HPV) in 2006 has provided an efficacious preventative technology that requires little in terms of health infrastructure beyond existing vaccination support structures (GlaxoSmithKline Vaccine HPV Study Group et al., 2009; Villa et al., 2006). Infection with HPV is linked not only to cervical cancer, however, but also anogenital warts and cancers of the oropharynx, anus, vagina and vulva, and penis. As such, the reduction in the global burden of disease attributable to widespread HPV vaccination would be even higher than the estimates of avoidable cervical cancer alone (Jemal et al., 2013). However, it is evident that the availability and feasibility of these interventions for prevention, screening, or treatment have not been enough to raise the issue of cervical cancer to a higher position on the global health agenda.

Policy agendas, however, are set through political processes, and not simply through evaluations of burden or distribution of disease, despite what the field of public health might aspire to achieve (Baltussen & Niessen, 2006; Murray & Lopez, 1997; World Bank, 1993). The aim of this article, therefore, is to apply a policy-analysis lens to explore potential reasons why cervical cancer may not currently feature highly on the global health agenda and consider the factors that may facilitate policy attention in the future. To do this, we utilise four established policy-analysis frameworks that have in recent years been applied to questions of international health priority setting: John Kingdon's (2003) ‘multiple streams’ model; Hall, Land, Parker, and Webb's (1975) model of ‘Legitimacy, Feasibility, and Support’; Shiffman and Smith's global health policy framework (2007); and finally Geneau et al.'s modified ‘political process model’ (2010) for chronic diseases. Each of these frameworks provides slightly different, although overlapping, perspectives that we use to reflect on the case of cervical cancer on the global agenda. Our analysis further allows us to discuss the appropriateness of these policy-analysis models for the specific nuances of *global* health agenda analyses, while also leading to conclusions regarding the key factors that cervical cancer advocates may wish to consider in pressing for greater global attention to the issue.

## Global health priority setting?

Political agenda setting is typically understood as the process undertaken by a decision-making authority in allocating attention and time among a variety of policy needs (Jones, 1994). At a national level, policy making revolves around the activities of a sovereign entity (i.e. the state), including a finite number of actors involved within it (Fidler, 2007). This typically provides a clearly delineated realm of activity, which has been studied thoroughly in the context of national health policy decision making, especially in low-income countries (Youngkong, Kapiriri, & Baltussen, 2009). However, priority and agenda setting for *international* or *global* policy is a much more nebulous concept, as Reich (1995, p. 489) argues, due to the lack of a ‘well-bounded institutional sphere’ such as a national legislature or ministry. The current global health decision-making environment has been described as having a large number of state, non-state, and multilateral actors; a lack of clearly defined decision-making institutions or formalised rules; and a lack of standardised accountability mechanisms between influential global actors and local citizens (Buse & Walt, 2000; Fidler, 2007; Gostin, 2007; Kickbusch, 2000).

An important result of this situation is that there is particular dynamism, or a lack of consistency, in the shape and influence of the institutional bodies involved in global health debates. Within a nation-state, one can usually take for granted some continuity in the importance and role of the Ministry of Health or the legislature from one health decision to the next. Yet, at a global level, there is less confidence that the institutional forms will persist. The WHO may appear to be an exception, as it is often thought of as a long-standing authority on global health matters, but there is growing recognition that the WHO is increasingly limited in its policy influence, as only one of many actors shaping global health debates (Mackey & Liang, 2013; Sridhar & Gostin, 2011).

In light of these realities, there may be arguably less practical use (for the purposes of global health planning, at least) in analysing the determinants of single-policy decisions made within individual international health institutions. Rather, recent analyses have instead investigated the factors that shape global health priorities more broadly, developing and applying frameworks of agenda setting from the field of policy analysis to guide investigation. Shiffman and Smith (2007), for instance, specifically developed a global policy-analysis framework to explore why the Safe Motherhood Initiative launched in 1987 was still in early stages 20 years later. The framework examines four determinants of political priority: the power of actors, the power of ideas, political contexts, and characteristics of the issue. This framework has subsequently been used by Shiffman (2010) to examine the issue of newborn survival, and by Tomlinson and Lund (2012) to explore where mental health sits on the global agenda. Reich (1995), alternatively, employed Kingdon's multiple streams model of agenda setting (2003) to explore the differences between child health and adult health on international agendas. In Kingdon's model, policy change occurs at favourable moments when policy problems are apparent, policy solutions are available, and political will is in place. The model further discusses the importance of ‘policy entrepreneurs’ who take advantage of opportune political moments (or ‘policy windows’) when these conditions are met (Kingdon, 1998). A third established policy-analysis framework often used to understand agenda setting at national levels is that of Phoebe Hall et al.'s (1975), which explains that issues get on a government's agenda when they have high levels of *legitimacy, feasibility*, and *support*. Hall et al.'s model was expanded by Bird et al. (2011) to explore the priority given to mental health across multiple African countries. Lastly, Geneau et al. (2010) recently used a ‘modified political process model’ to describe how to elevate chronic diseases on the global agenda, presenting three key recommendations: reframing the debate, identifying and creating political opportunities, and mobilising resources.

Each of these approaches attempts to map out key factors that facilitate or hinder policy change, so as to make recommendations to improve the placement of an issue on a policy agenda. As such, these frameworks can all provide complementary insights into the case of cervical cancer to explore why it may not have received global health attention in the past, and they can point to potential strategies to increase the attention cervical cancer receives in the future. Rather than selecting a single framework to answer this issue, this paper adopts the approach described by Cairney (2007), which utilises different policy-analysis frameworks as ‘lenses’, each of which can provide some insights into the case study at hand. Table 1 provides a brief summary of the four frameworks utilised here.

**Table 1.** Summary table.

Framework	Developed from	Key components	Examples where applied to international health
Shiffman and Smith (2007)	Investigation of global maternal health priority-setting	1. Power of actors 2. Power of ideas 3. Policy context 4. Characteristics of issue	Safe motherhood initiative (Shiffman & Smith, 2007), newborn survival (Shiffman, 2010), mental health (Tomlinson & Lund, 2012)
Geneau et al. (2010)	Adaptation of the ‘political process model’, used to study social movements, mostly in the US	1. Reframing the debate 2. Identification and creation of political opportunities 3. Mobilisation of resources	Global attention to chronic disease (Geneau et al., 2010)
Hall et al. (1975)	Social policy model derived from case studies in the United Kingdom	1. Legitimacy 2. Feasibility 3. Support	Mental health in African countries (Bird et al., 2011), reproductive health services in conflict settings (Palmer & Zwi, 1999)
Kingdon (2003) (originally published 1984)	General policy change framework developed from US policy making	1. Problem stream 2. Policy stream 3. Politics stream 4. Policy entrepreneurs	Child and adult health (Reich, 1995)

It is of course worth recognising that these frameworks, as detailed as they are, represent only a small sample of the broader body of work in the social and political sciences attempting to understand why and how some political issues receive attention while others do not (Burstein, 1991; Grindle & Thomas, 1991; Hilgartner & Bosk, 1988; Hogwood & Gunn, 1984; Moran, Rein, & Goodin, 2008). Sociological works, for instance, have questioned how issues get constructed or how society recognises issues as ‘problems’ to begin with (Gusfield, 1984; Hilgartner & Bosk, 1988), while John (1998) has classified the main approaches commonly taken to analyse public policies as: institutional approaches, group and network approaches, rational choice theory, and ideas-based approaches. While an extensive body of work exists for each of these, John argues that a synthesis of approaches is needed (presenting Kingdon's multiple streams model as one possible candidate to do this; John, 1998). Theories from any one approach may be useful, but do not explain policy change and agenda setting on their own. As such, each of the frameworks utilised here incorporates multiple explanatory elements in their application to global health policy analysis. In the results section following, we thematically look at how the case of cervical cancer fits into the main issues raised by the frameworks, in order to draw out insights that may explain cervical cancer's current place in the global health landscape, as well as to identify potential issues advocates may wish to address in future considerations.

## Results

### Problems and solutions

The first common element across all frameworks is recognition (or an explicit statement) that the problem must be known and that solutions must be available to engender prioritisation. As the introduction has shown, there is significant macro-level data on the burden of cervical cancer; the inequitable global distribution of cervical cancer deaths; and well-established prevention, screening, and treatment technologies. That said, Sitas et al. (2006) and Ferlay et al. (2010) have noted that local-level cervical cancer indicators are lacking in much of sub-Saharan Africa due to limitations in systematic reporting, cancer registries, and information collection. It may be difficult to raise cervical cancer as a priority issue if national actors are not aware of the scale of the problem in their home country.

Furthermore, having knowledge of cervical cancer rates and of prevention and treatment options may be insufficient if there is the perception that the existing medical infrastructure and resources are unable to provide treatment, as is often assumed in low-and middle-income countries. Cervical screening coverage in sub-Saharan Africa, for instance, ranges from 2% to 20% in urban areas and from 0.4 to 14% in rural areas (Louie, de Sanjose, & Mayaud, 2009). There are few large-scale initiatives for cytology screening, visual inspection, or testing. While newer technologies have been found to be more cost-effective, there still may be resistance or simply inertia leading to slow uptake of these technologies.

The absence of cytology programmes in many African nations may also be due to a perceived inability to treat established or advanced cervical neoplasms (Sankaranarayanan et al., 2001; Sitas et al., 2006). A current lack of capacity, however, should not necessarily serve to prevent action. As noted in the introduction, screening and cryotherapy treatment alone are expected to provide significant benefits with relatively low infrastructure or training requirements. While infrastructural challenges remain in treating more advanced cases of cervical cancer, we note that similar arguments were made about the inability to provide antiretroviral drugs for HIV treatment in low-income settings until operational research illustrated the feasibility of doing so in resource-constrained areas (Farmer et al., 2001), and donors dedicated resources to building this capacity.

Evaluation of pilot programmes may therefore be particularly useful to illustrate how low-income countries can, in fact, operationalise screening and treatment to help change any perception that it is infeasible or not worthwhile. Initial pilot projects to establish VIA in India, Uganda, Peru, and Vietnam, for example, were conducted by the agency Program for Appropriate Technology in Health (PATH; Cervical Cancer Action, 2012) and can be used as examples elsewhere. The HIV experience further shows that when international priority is given to an issue (i.e. antiretroviral treatment), then investments may follow to provide the required infrastructure (Yu, Souteyrand, Banda, Kaufman, & Perriëns, 2008). Indeed, there have been efforts to implement national or regional cervical cytology screening programmes in Latin American countries since the 1970s (Sankaranarayanan et al., 2001, p. 954), which no doubt would have required developing necessary infrastructure at the time as well.

In contrast, vaccination for prevention may not require significant infrastructural development, but the high cost of the HPV vaccines may undermine the feasibility of this as a solution. However, HIV again provides a clear case of how political will can lead to reductions in prices of patented drugs for countries that otherwise could not afford them, and how generic manufacturing may also be possible. Indeed, the Global Alliance for Vaccination and Immunisation (GAVI) has recently included the HPV vaccine in its immunisation package and negotiated a price of US$5 with Merck in order to subsidise vaccines for eligible low-income countries (GAVI Alliance, n.d.). Further work is being done to form public–private partnerships to share costs and make advanced market commitments to subsidise vaccines (Louie et al., 2009). Unfortunately, middle-income countries, which currently do not qualify for GAVI-negotiated prices, may still face challenges of vaccine affordability (Wilson, 2010), but there are clear indications that cost and infrastructure concerns may be changing.

In summary, policy frameworks commonly note the necessity of both a clear problem and a viable solution to raise the priority of an issue. While there are clear indicators of a cervical cancer problem in low-income countries, data may be lacking at national, regional, and local levels to actively mobilise national stakeholders in global forums. Moreover, we contend that clarifying the nature of the problem and the potential solutions available may not be sufficient to lead to global health prioritisation on its own. Indeed, for all the policy-analysis frameworks considered, the recognition of a problem and the availability of a solution represent only a starting point. As such, these frameworks have specifically been developed to address the factors at play when problems (with solutions) are not featured on political agendas.

### Power, networks, and resources

A second commonality across the frameworks is that each in some way conceptualises the importance of the level of support for an issue in terms of actors and resources. Shiffman and Smith's (2007) first category explicitly addresses the power of actors, which is said to derive from a cohesive policy community, strong civil society mobilisation, and a widely embraced leader. This is closely linked to Geneau et al. (2010), who refer to mobilisation of resources (financial, human, or technical) as elements of an actor's power. The importance of the power of actors in support of issues is less explicit in the Hall et al. and Kingdon frameworks. This is potentially due to those frameworks’ origins in studying national policy making, where a single authority (i.e. the state) holds the power to change policy. Instead, Hall et al. (1975) refer to ‘support’ for an issue by the decision-making authority and Kingdon's ‘politics’ stream refers to political will – both of which are only indirectly linked to the power of different interest groups who may be trying to shape an agenda. For global health policy, however, the more explicit attention to power appears warranted, as the context is typified by a multiplicity of actors in continual competition to set global agendas.

In terms of cervical cancer, there appears to be a moderate level of human resources and networks dedicated to the issue globally. The formation of multiple cervical cancer alliances in the past decade gives evidence of a collaborative policy community, with potentially more cohesion than the Safe Motherhood Initiative in 1987, which was reported to face a divided policy community from its inception (Shiffman & Smith, 2007, p. 1373). Actors have created partnerships like Alliance for Cervical Cancer Prevention and Cervical Cancer Action, which try to increase global attention and work to share resources (Sherris et al., 2009). There is usually broad consensus among these actors on recommended interventions for prevention, screening, and treatment (Cervical Cancer Action, 2012; Franco et al., 2006; Sauvaget et al., 2011), although the lack of consistent political resources dedicated to the issues appears to show a lack of systematic global or national concern for the issue. Where there are controversies, they typically revolve around the financing of the HPV vaccine or whether to address cancers and other NCDs in the face of a perceived unfinished agenda for communicable diseases (Adeyi, Smith, & Robles, 2007).

There is also a range of other central elements of actor power, such as the mobilisation of wider civil society actors and key influential leaders. While civil society efforts have grown, the cervical cancer advocacy community has yet to rally around a policy champion (or champions) strategically placed to promote cervical cancer on the global health agenda, with little evidence of key influential leaders highlighting cervical cancer at UN High Level or post-2015 meetings, for instance. However, some international organisations have promoted screening and treatment. For example, (as noted) PATH has launched many VIA pilot projects in lower-income settings (Cervical Cancer Action, 2012); Pan-American Health Organisation created a Revolving Fund to offer HPV vaccines in Latin America at a reduced price of US$12 a dose; GAVI pledged to provide HPV vaccines, contingent on negotiations with manufacturers, for 28 million girls by 2020 (GAVI Alliance, n.d.). Interest groups in African countries have mobilised to spread awareness about cervical cancer screening. In Tanzania, for instance, First Lady Mama Salma Kikwete has been a dedicated patron of the Medical Women's Association of Tanzania by raising awareness about cervical cancer and attracting donors to support their efforts (Ngiloi, 2006). There has been some pan-African advocacy as a result of the combined efforts of First Ladies of several countries who have started a Forum of African First Ladies Against Breast and Cervical Cancer and held several conferences under the banner ‘Stop Cervical Cancer in Africa’ (www.stopcervicalcancerinafrica.org). Cervical cancer has also received funding from charitable foundations such as the Bill & Melinda Gates Foundation, which awarded US$50 million to Alliance for Cervical Cancer Prevention in 1999 and US$10 billion to ‘Decade of Vaccines’ to research, develop, and deliver vaccines to the world's poorest countries (Bill & Melinda Gates Foundation, 2000, 2010). More recently, the Pink Ribbon Red Ribbon partnership pledged US$75 million to combined breast and cervical cancer efforts (Rosenthal, 2011).

While these examples appear to illustrate growing human and financial resources for cervical cancer, challenges still remain. Many of these initiatives have not mobilised those affected by cervical cancer in an extensive way, typically reflecting top-down or outsider-led mobilisation. As a whole, cancer has also not received as much priority as infectious diseases. Even within the realm of cancer, there is a risk that cervical cancer may be overshadowed on national agendas. Perhaps, this is best illustrated by Reichenbach (2002), who demonstrated how public mobilisation around breast cancer placed it higher on local agendas in Ghana despite the higher burden of disease and cost-effectiveness analysis pointing to cervical cancer as the logical women's health priority in that country.

Finally, there is also the persistent challenge faced in achieving priority for women's health issues, given the disempowerment of women generally in many countries, and the low emphasis placed on women's health concerns. Maternal mortality, however, again provides a useful comparator to show that it has been possible in the past for a women's health issue to be taken up in global calls such as the Millennium Development Goals (WHO, 2009b). Yet, some might argue that it took a disproportionate amount of time and effort to raise this issue on the global agenda, in part because of the systematic underrepresentation of women or structural bias against women's health in national and global policy circles (fundamentally, a lack of power and resources). Indeed, the safe motherhood movement was launched in 1987 and it took over a decade for maternal mortality to be taken up as a global priority (Starrs, 2006). As such, the relative power and influence of groups prioritising women's health will no doubt remain a critical component in shaping the place of cervical cancer on future global health agendas.

### Ideas, framing, and issue presentation

A third area the frameworks point to is conceptualisation or framing of the issue. There is a large sub-field of policy analysis that looks exclusively at framing and discourse to explain why and how particular issues arise on the policy agenda (Fischer, 2003, 2007; Schön & Rein, 1994). When applied in public health work, typically framing is taken to represent the key issues associated with a health condition, or the other political issues it is linked to in the broader discourse around it. Shiffman and Smith refer to the power of ideas as key to shaping how an issue is framed, and they point to the famous ways in which HIV/AIDS has been framed at different times as a security issue, a human rights issue, a development issue, and so forth. One can similarly see this kind of framing with the safe motherhood descriptions of global maternal deaths equating to a jumbo jet full of childbearing women crashing each day (WHO, 1986). This language shifts the conceptualisation from one of bare statistics to an emotional association of the tragic and avoidable loss of mothers. Hall et al. and Kingdon's models do not address framing explicitly, but Geneau et al. (2010) calls for ‘reframing the debate’ as one of their three key recommendations to shape global health agendas so as to increase attention to chronic diseases.

A review of current literature on cervical cancer points to opportunities to reconsider how the issue is framed and to reflect on what other agendas it might be linked with. Much of the identified literature reviewed has primarily framed cervical cancer as a technical issue, using the language of epidemiology and public health, most likely for the benefit of Ministers of Health and clinical or academic researchers. As noted in the introduction to this paper, however, technical facts on the burden of cervical cancer, the inequities in that burden, and the availability of treatment have not been enough to raise the issue on the global agenda. However, we identified three ways that, in the past few years, cervical cancer has been linked to other issue agendas, which serves to help raise its priority or increase attention to the condition.

First is that a number of cervical cancer stakeholders have begun to frame the disease under the umbrella of NCDs (Cervical Cancer Action, 2012). Lately, NCDs have emerged as an increasing global health priority – as evidenced by the recent UN High Level Meeting on NCDs (UN General Assembly, 2011). Undoubtedly, there are emerging calls for greater political attention to NCDs as a broader group of conditions (Geneau et al., 2010; WHO, 2011a), perhaps due to growing recognition of ageing populations, rising incomes, and increasing exposure to risk factors that contribute to NCDs (Adeyi et al., 2007). While framing cervical cancer as an NCD may also lead to it having to compete with a number of other health issues within this category (Beaglehole et al., 2011), it does ensure that cervical cancer does not get left out altogether from the emerging global policy agenda focus on NCDs.

Second, activists like Princess Nikky of Nigeria and other African First Ladies have framed cervical cancer as it is relates to women's rights and women's health. This approach attempts to move outside the epidemiological burden of cervical cancer to link it to a broader social priority of gender equality, human rights, and health. To date, however, these efforts seem to have provided limited changes in the conceptualisation of cervical cancer on the global health agenda.

Finally, there have been examples of cervical cancer being linked with HIV/AIDS, due to the fact that the risk for cervical cancer is much higher in HIV-positive women (Engels et al., 2008; Mbulaiteye et al., 2006). Although the risk for many other conditions (including maternal death or other cancers) is also much higher in HIV-positive individuals, this strategy appears to have been successful in permitting the US President's Emergency Plan for AIDS Relief (PEPFAR) and the Joint United National Programme on HIV/AIDS (UNAIDS) to serve as key supporters of the Pink Ribbon Red Ribbon partnership, which works to combat cervical and breast cancer in Africa. According to PEPFAR, support for this partnership serves to ‘expand the availability of vital cervical cancer screening and treatment – especially for HIV-positive women, who face a dramatically heightened risk of cervical cancer’ (President's Emergency Plan for AIDS Relief [PEPFAR], 2011).

The framing of cervical cancer is in many ways unique, as it deals with two very differently conceived issues: (1) prevention of HPV, a sexually transmitted infection (STI) and (2) prevention and treatment of cases of cervical cancer, an NCD. STIs are often politically charged due to the moralisation of sexual behaviour and stigmatisation of sexual infections (Gilmore & Somerville, 1994; Lichtenstein, 2003; Mulholland & Van Wersch, 2007). Conversely, cervical cancer is often associated with older women who lack access to health services such as early detection and treatment. Advanced cases of cervical cancer can be embarrassing or stigmatising for the individual, but there is less stigmatisation related to blame or moralisation of the individual's behaviour.

As cervical cancer so uniquely sits on two very differently conceptualised issues, advocates may consider how their framing of cervical cancer and its prevention mechanisms evoke different issue characteristics (e.g. whether the condition is easily subject to stigma), which in turn support or hinder its placement on global agendas. The debates around HPV vaccination in cervical cancer literature appear to show that these nuances are already being recognised. For instance, Merck's HPV vaccine Gardasil™ has strategically been constructed as a vaccine against ‘cancer’ rather than against an STI (Epstein & Huff, 2010). Gardasil HPV vaccines are also marketed as ‘a woman's right, choice, and duty for health security’ (Graham & Mishra, 2011, p. 511) thereby invoking a women's rights frame, although potentially diluting the scope of vaccine effectiveness for males. Although the American Academy of Pediatrics recommends that boys receive the vaccine between ages 11 and 12, there is low awareness regarding benefits of male vaccination and subsequently, low rates of vaccination among American men (Stupiansky, Alexander, & Zimet, 2012). Further policy discussions will need to include males in the HPV and cervical cancer discussion and keep aware of these framing considerations.

### Key moments

A final important consideration derives from one of the most important contributions of Kingdon's (2003) multiple streams framework to the field of policy analysis. This framework not only identifies factors that must be in place for policy change to occur but also specifically highlights the importance of timing. The most critical point in time is when Kingdon's multiple streams ‘converge’ to open a ‘policy window’, an opportunity for policy change. Kingdon further describes the importance of policy entrepreneurs who can recognise and take advantage of these windows of opportunity, which overlaps with Geneau et al.'s (2010) consideration of opportunities for political change. Shiffman and Smith (2007) also use the language of policy windows in their conceptualisation of the policy context.

Yet, while the notion of policy windows has been a widely influential concept in studies of *national* policy change, Geneau et al. and Shiffman and Smith's frameworks, having been developed specifically for global health issues, provide particular insights into the type of opportunities that are most relevant for global agenda setting. Meetings of multilateral institutions, such as the World Health Assembly (or even the UN General Assembly), and international conventions are particularly emphasised in these frameworks, with emphasis on meetings that lead to widely embraced international agreements (e.g. the Millennium Development Goals).

A recent global policy window of this kind was the UN High Level Meeting on NCDs in September 2011. Two years earlier, four organisations formed the non-communicable diseases (NCDs) Alliance to make recommendations for the UN High Level Meeting, with the Union for International Cancer Control as the lead organisation representing cancer (Beaglehole et al., 2011). This Alliance released a document in July 2011 with goals and priority targets that include giving priority to early detection, screening, and diagnosis of cervical cancer and other cancers (Beaglehole et al., 2011). The African First Ladies Forum used this UN meeting in September 2011 to draw attention to cervical cancer as an NCD (Okore, 2011). At a side event of the UN meeting, the Ambassador of Malawi further made a speech advocating the need to make cervical cancer a global priority for prevention (Bowler, 2011). In South Africa, the national cervical screening programme that began in 2000 was found to have screened 30.8% of women in the Johannesberg Metro District by 2008 (Jassat, 2010). Other African Ministers of Health have emulated such programmes, with Rwanda implementing its first national cervical cancer prevention programme in 2011 (Binagwaho, 2012). These are important steps for the cervical cancer agenda, and they suggest that future meetings of this nature, perhaps around the post-2015 Millennium Development Goals, may prove to be opportunities to influence global policy agendas.

## Discussion

This article has reflected on the case of cervical cancer on the global health agenda, using a variety of policy frameworks to understand why cervical cancer may have not received the political attention it might otherwise merit, considering the burden of disease carried by low-income countries and the preventability of cervical cancer mortality. We began by pointing to the increasingly cost-effective ways to prevent, screen, and treat cervical cancer (through vaccination, VIA, and cryotherapy). We then noted the specific challenges of agenda setting at a global level, and how this may differ from national decision-making processes.

From this point, we then analysed the cervical cancer question through the application of four policy agenda-setting frameworks that have been applied to health policy issues at national and global levels in the past. The frameworks have a number of overlapping insights that apply to the case of cervical cancer, including the need to have a clearly identified problem and solution; networked actors with suitable resources (power) to promote the issue; appropriate framing of the issue; and key moments in which to press for change. Using a combination of policy-analysis frameworks has proven to be particularly useful, as they each have complementary strengths to bring to the analysis. Their application in this case, however, also allows reflection on their appropriateness to analyses of global health policy making more broadly.

The Hall et al. (1975) model, developed from consideration of a single state's action, as such proves more difficult to apply to the global priority questions around cervical cancer. The international priority-setting challenges that arise from having multiple actors with differing motives and priorities appear difficult to capture. For example, Hall et al.'s model examines ‘administrative capacity’ as a component of feasibility, which has much less clear meaning at the global level. Bird et al. (2011), however, adapted the model to include a number of additional global health concerns, notably including the importance of stigma of health issues. If cervical cancer is associated with promiscuity and poor hygiene, and people are blamed for their condition, this stigma may make it even harder to mobilise individuals affected by the condition and make it harder to generate the political will necessary to raise it on agendas.

Kingdon's model, in contrast, was found to be most useful and unique in its explicit consideration of the time dimension, with Kingdon himself noting that policy windows stay open only for short periods (Kingdon, 2003). The recent UN High Level Meeting on NCDs in September 2011, for instance, provided one such window to raise cervical cancer issues. Kingdon's framework also adds the role of key policy entrepreneurs as central to the explanation of policy change. In global policy processes, there are a lack of formal institutional bodies and rules for global priority setting, and a proliferation of actors involved. As such, the importance of key policy entrepreneurs who are linked into appropriate networks (which may be constantly changing) may be particularly important.

Finally, the importance of Shiffman and Smith's and Geneau et al.'s frameworks lies in their explicit focus on global policy agenda questions and opportunities that can be targeted by health policy advocates. Moreover, they both bring the notion of framing to the forefront. While there has been little discursive framing analysis of global health issues, the diversity of actors and the sheer magnitude of cultural and ideological heterogeneity at the global level may increase the relevance of framing of issues. It is perhaps noteworthy that none of these frameworks explicitly addresses the concept of policy transfer and learning from abroad (Dolowitz & Marsh, 2000). Although this analysis focuses on the global policy agenda, policy-transfer theories may provide additional insights in understanding individual country decision making and the rate at which nations take up global recommendations. Such studies, for example, may examine whether there is stigma associated with being the first or last country to address a particular issue.

## Recommendations and conclusion

Cervical cancer and public health advocates will no doubt be interested in an analysis such as this to identify lessons they can use to help promote cervical cancer prevention and treatment on global agendas. Perhaps, it is most critical for these actors to recognise that policy agendas are not set by appeals to evidence of health burden or distribution alone. Nathanson (2007) describes health policy making as a process of social change, rather than a rational assessment of health need. In this vein, she sees shifts in health policy agendas as the result of social movements, the success of which is based on ‘(1) the articulation of a socially (as well as scientifically) credible threat to the public's health, (2) the ability to mobilise a diverse organisational constituency, and (3) the convergence of political opportunities with target vulnerabilities’ (Nathanson, 1999, p. 421). Similarly Goodin, Rein, and Moran (2006) refer to policy making as a process of ‘persuasion’. For public health advocates wishing to promote a particular agenda, they may wish to reconsider their role as participating in a social movement and involved in a process of argumentation and persuasion on what is important in the global health and development sector, rather than simply assuming global agendas will eventually reflect epidemiological realities.

One approach towards working from this perspective may be to expand cervical cancer framing from technical terms alone to a human rights and social justice frame. Such messages may be more resonant with policy makers and the public alike when presentation of statistical burden-of-disease information is unconvincing, or when the marginalisation of the group affected (poorer women in many cases) means their needs are rarely seen as an important public health concern. Global health equity-based language, however, has been a common strategy for other issues, ranging from kidney disease (Dirks et al., 2005) to the social determinants of health (Marmot, Allen, Bell, & Goldblatt, 2012). Learning from the framing and strategies taken around a health-equality model may be one strategy to help legitimise the prevention and treatment of cervical cancer as a higher priority in low-income settings.

For cervical cancer to achieve higher global priority, advocates must strategically mobilise resources and frame the debate to gain widespread support, but they critically must press for policy change when particular policy windows are open. The increasing global attention to NCDs in the post-2015 agenda provides a number of opportunities to highlight the issue of cervical cancer and integrate it into new agendas. Governments will no doubt need to invest in a range of health systems elements to successfully manage chronic diseases and this can help overcome arguments that it is too difficult or expensive to manage cervical cancer. However, there is also a vaccination agenda, less discussed in cervical cancer literature, into which HPV vaccination programmes could conceivably be integrated. HPV vaccination has led to a high level of contestation, however, due to the sexually transmitted nature of the virus, which may hinder progress in this area (Graham & Mishra, 2011). But it remains an important strategy to pursue, particularly for advocates concerned with the range of HPV-related morbidities, not just cervical cancer.

There are also areas that need further research and pilot work; however, much of that is practical or operational in nature, such as investigating how the framing of cervical cancer affects its support in different settings; having negotiations on reducing costs of vaccinations for poorer countries; and conducting feasibility studies of pilot programmes for screening and treatment in sub-Saharan Africa. There are definite ripples of advocacy occurring in the sphere of international cervical cancer policy, which in many ways appears to be in a better position than the safe motherhood agenda when it was first launched. Safe motherhood, however, struggled for a long time to gain global attention. By taking advantage of the insights that these policy frameworks have shown, actors may be able to reduce the time it takes to mobilise global action and address the challenge of cervical cancer in low-income settings.
